# Regional prevailing wind directions cannot explain the global east facing of mature sunflower inflorescences: testing a hypothesis using wind data

**DOI:** 10.1007/s00425-025-04794-y

**Published:** 2025-08-09

**Authors:** Enikő Rajna, Tamás Weidinger, Gábor Horváth

**Affiliations:** 1 Department of Biological Physics, Institute of Physics and Astronomy, ELTE Eötvös Loránd University, Pázmány Sétány 1, 1117 Budapest, Hungary; 2 Department of Meteorology, Institute of Geography and Earth Sciences, ELTE Eötvös Loránd University, Pázmány Sétány 1, 1117 Budapest, Hungary

**Keywords:** East facing, *Helianthus annuus*, Meteorology, Plant-environment interaction, Prevailing wind, Sunflower

## Abstract

**Main conclusion:**

Our finding that mature sunflower inflorescences face east independently of the prevailing wind direction shows that the wind is an unimportant environmental factor in the orientation of sunflower heads.

**Abstract:**

According to a biomechanical hypothesis, the constant east facing of mature sunflower inflorescences may be caused by the local average prevailing wind blowing nearly from west to east, because such winds could force the sunflower head to turn approximately eastward due to the torque (turning-moment) exerted by the air drag. In this work we test this hypothesis, using the wind data of Hungary, Europe and the USA originating from the ERA5 MONTHLY database averaged for the May-August breeding-season of sunflowers and the periods 2014–2023, 2004–2023, 1974–2023 and 1940–2023. For the longest averaging period 1940-2023, we found that the percentage *f* of regions with average prevailing wind direction α pointing nearly to the geographical east α = 0° ± 15° in the area of Hungary, Europe and the USA without larger water surfaces and mountains is *f* = 2.6 ± 5.8%, 11.4 ± 5.0% and 13.7 ± 2.8%, respectively. This means that the average prevailing wind could turn approximately eastward (α = 0° ± 15°) the sunflower inflorescences only in very small parts of the three studied regions. Since the majority of mature sunflower inflorescences orient everywhere nearly to the geographical east, the hypothesis in question is not supported by our findings, because the mentioned meteorological prerequisite is not fulfilled.

**Graphical abstract:**

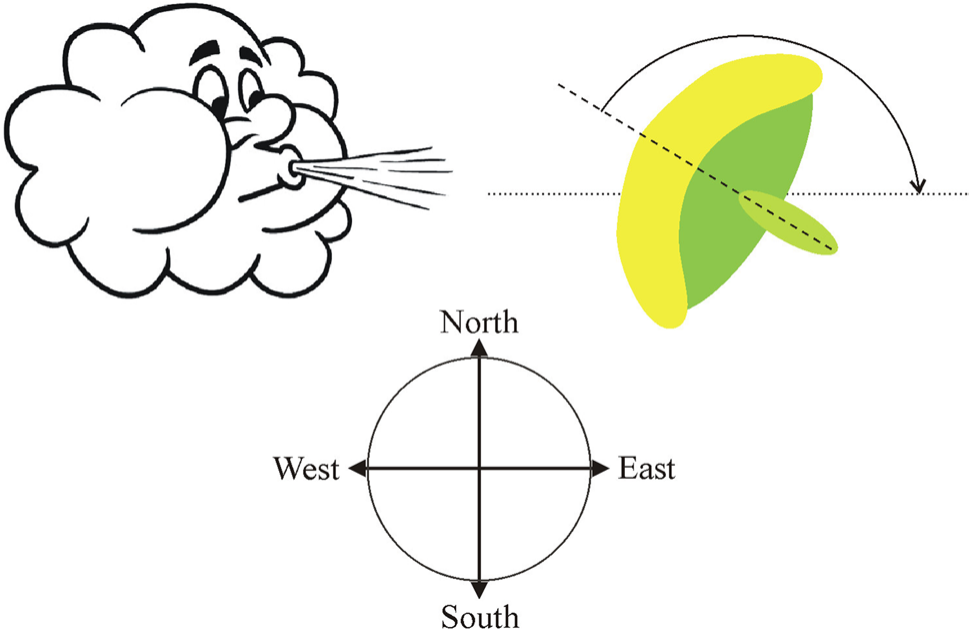

## Introduction

The normal vector of immature (non-flowering) heads and young leaves of sunflowers (*Helianthus annuus*) follow the sun in the sky (Darwin and Darwin 1897): after sunrise the head turns from east to west, while after sunset it turns back from west to east (Lang and Begg 1979). From the beginning of flowering, the mature inflorescences do not follow the sun, their heliotropism ceases, and they face constantly nearly to the geographical east (Takács et al. 2022a). After head and leaf formation, the absorbed sunlight and photosynthesis serve the production and maturation of seeds. One of the advantages of east facing of *Helianthus annuus* is that eastward oriented sunflower heads have maximal number and mass of kernel-filled seeds (Takács et al. 2022b).

**Fig. 1:**
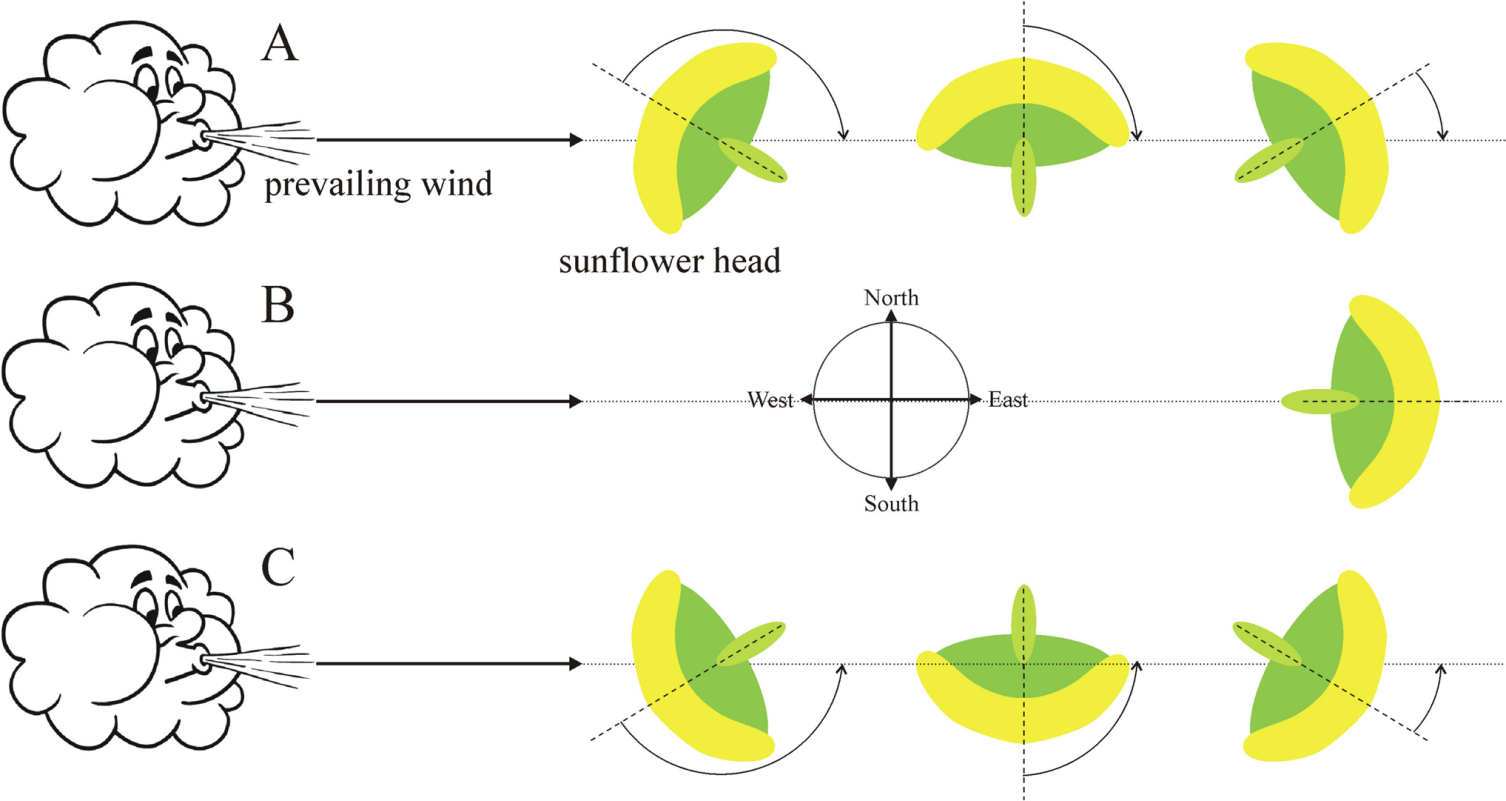
**A**–**C** Illustration of the biomechanical hypothesis that the local prevailing west-to-east wind direction may cause the east facing of mature sunflower inflorescences, because the torque exerted by air drag turns the head toward the wind direction (**A**, **C**), apart from the situation when the head’s symmetry axis (dashed line) is already parallel to the wind (**B**)

For the possible benefit(s) of the eastern orientation of mature sunflower inflorescences at least eight, not mutually exclusive explanations have been proposed, but only two of them were tested so far:

1. Decrease of the seed loss by seed-eating birds (Seiler 1997).
2. Reduction of heat stress at noon (Leshem 1977; Lang and Begg 1979).
3. Prevention of fungal attack by the greater reception of solar radiation in the early morning, which speeds up drying of morning dew (Lang and Begg 1979).
4. Maintenance of the viability and fertility of pollens by decreasing the heat stress in the afternoon (Ploschuk and Hall 1995; Seiler 1997).
5. Reduction of the temperature of sunflower heads (Lang and Begg 1979; Ploschuk and Hall 1995; Lamprecht et al. 2007).
6. Enhancing the attraction of insect pollinators (Lamprecht et al. 2007; Atamian et al. 2016).
7. Maximizing the light energy absorbed by mature sunflower inflorescences (Horváth et al. 2020).
8. Biomechanical effect of the local prevailing wind blowing from west to east (Horváth et al. 2024; Rajna 2024): The east facing of sunflower inflorescences may be caused by wind, if the local prevailing wind direction is eastern in the sunflower’s breeding-season. Then, the torque exerted by the wind-generated airdrag could turn the sunflower head toward east (Fig. 1). This hypothesis was proposed by Prof. Imre Derényi (Department of Biological Physics, Eötvös University, Budapest, Hungary) on the 29th Biophysical Congress in Budapest, on 31 August 2023, after the lecture given by Horváth (2023).

Horváth et al. (2020) gave a critical review of hypotheses (1)-(5). They showed that hypotheses (1), (2) and (4) are physically/meteorologically erroneous, hypothesis (3) may be correct but is experimentally not tested/validated, and hypothesis (5) is partly supported by the results of Takács et al. (2022b). In field experiments Horváth et al. (2024) showed that the pollinator visits of sunflower inflorescences in *Helianthus annuus* plantations are independent of head orientation. By this finding the wide-spread hypothesis (6) was refuted. On the other hand, using astronomical, meteorological and plant physiological data, Horváth et al. (2020) showed that sunflower inflorescences absorb maximum light energy if they face east and afternoons are cloudier than mornings, as is typical for eastern North America, the domestication region of *Helianthus annuus* (Blackman et al. 2011). This result corroborated hypothesis (7).

In this work we test the newest hypothesis (8): We study here where could the local average prevailing west-to-east wind direction in the sunflower’s breeding-season cause the east facing of mature sunflower inflorescences. The most important meteorological prerequisite of this is that in the breeding-season of sunflowers the prevailing wind should blow nearly from west to east in all regions of sunflower production, because all mature sunflowers face east. Further on in this work, the wind’s name refers always to the direction toward the wind is blowing. Thus, eastern wind means west-to-east direction of wind blowing toward east (from west), for example. The preliminary basis of our investigation is the diploma work of Rajna (2024). Using wind data of the ERA5 MONTHLY ReAnalysis (Hersbach and Dee 2016; Hersbach et al. 2020) in Hungary, Europe and the USA, we determined the area proportion *f* of different prevailing wind direction intervals averaged for the May–August breeding-season of sunflowers and the last 1, 2, 5 and 8.3 decades. Then, we focused on the west-to-east wind direction interval −15° ≤ α ≤  + 15°, where α is measured counter-clockwise from the geographical east (α = 0°). Obviously, the larger/smaller the value of the west-to-east wind frequency (*f*) in a given region, the more/less the wind data support hypothesis (8). We show here that wind data do not support this hypothesis, because the mentioned meteorological prerequisite is not fulfilled.

## Materials and methods

### ERA5 database

**Fig. 2:**
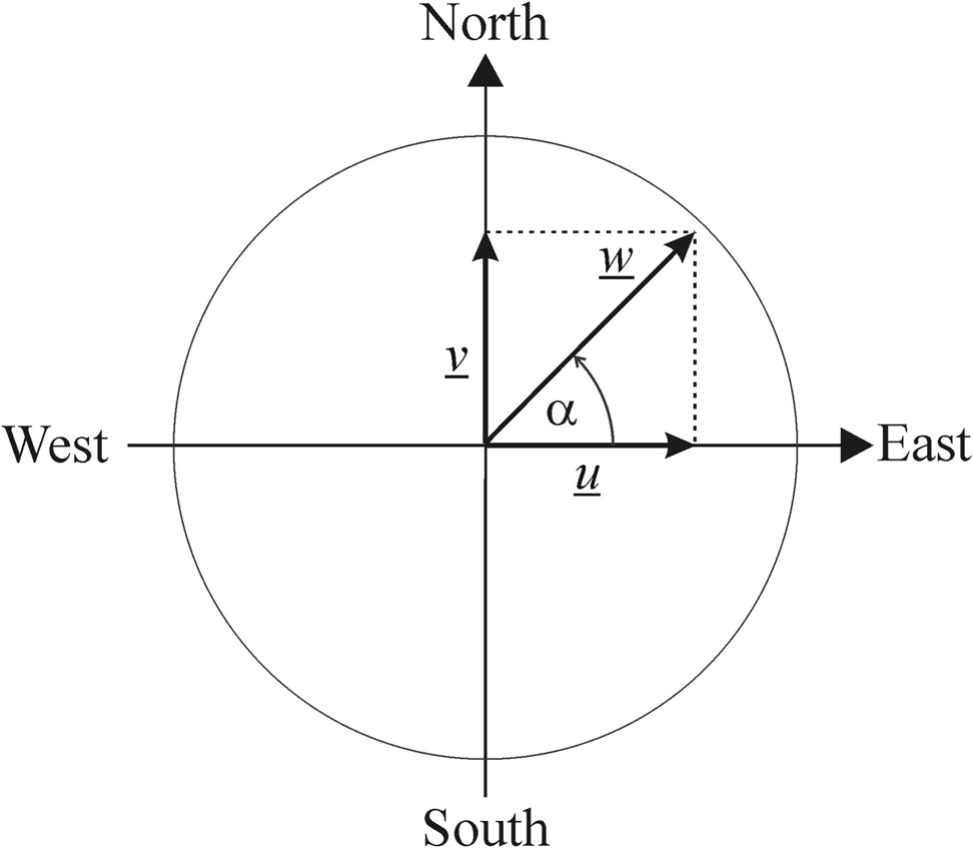
Orthogonal component vectors *u* and *v* of the wind speed vector *w* closing angle α counter-clockwise with the geographical eastern direction

For the determination of the average prevailing local wind direction in the breeding-season (May–June–July–August) of sunflowers in Hungary, Europe and the USA, we used the ERA5 MONTHLY database (Hersbach and Dee 2016; Hersbach et al. 2020; https://cds.climate.copernicus.eu/cdsapp#!/dataset/reanalysis-era5-single-levels-monthly-means?tab=form). ERA5 is the 5th generation ECMWF (= European Centre for Medium-Range Weather Forecasts) atmospheric reanalysis of the global climate. Using physical disciples and rules, it combines the meteorological model datas with observations originating from all over the world to obtain a globally complete and consistent dataset involving several decades. Using the hourly values of ERA5, the ERA5 MONTHLY provides aggregated values for every month of the year for the following meteorological variables: (i) air- and dewpoint temperatures measured at a height of 2 m above the ground, (ii) total amount of precipitation, (iii) average air pressure at sea level, (iv) ground air pressure, (v) orthogonal component vectors *u* and *v* of the wind speed vector *w* measured at a height of 10 m above the ground (Fig. 2).

**Fig. 3:**
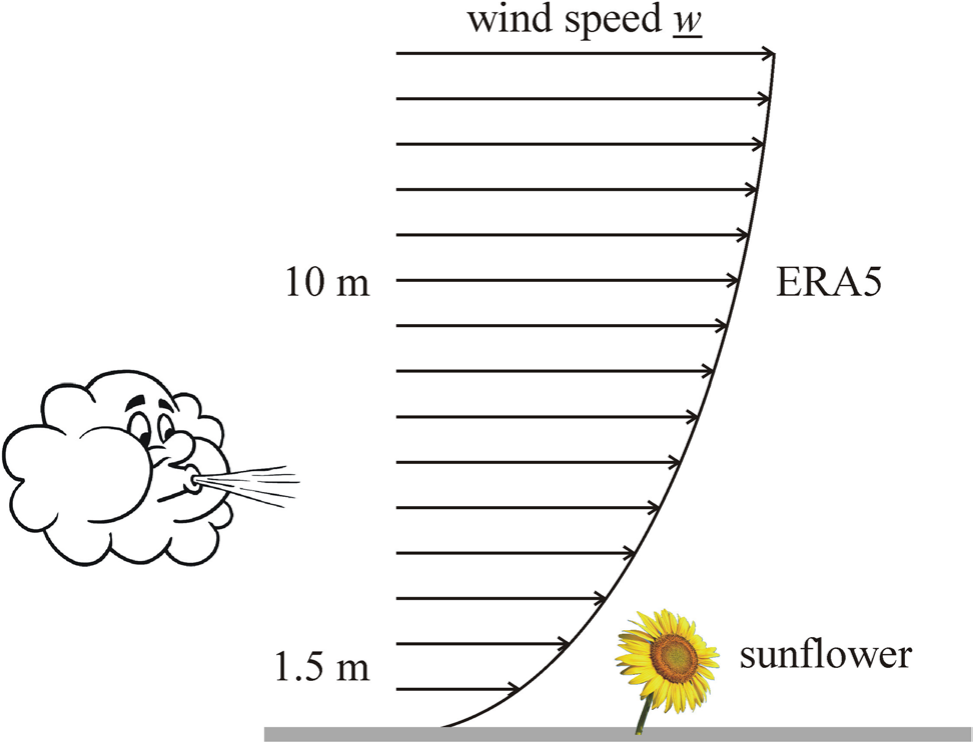
Qualitative illustration of the vertical change of the horizontal wind speed *w* represented by horizontal arrows above the ground (grey). Although the ERA5 wind data originate from a height of 10 m and the average sunflower height is only ~ 1.5 m, the direction of the wind speed vector *w* usually does not change within 10 m (Foken 2017)

In principle, we are interested in the prevailing local wind direction at the average height of ~ 1.5 m of sunflower heads (Seiler 1997). Since detailed wind data at this height are not available in the ERA5 database, we assumed that the wind direction at heights 1.5 and 10 m are the same. This assumption is sound, because within the height range of 1.5–10 m the wind direction does not change considerably, since this is in the surface layer with practically constant turbulent fluxes and wind direction (Stull 1988; Foken 2017). In contrast to the wind direction, the magnitude of wind speed increases with height (Fig. 3) depending on the surface roughness and atmospheric stability (Arya 2001; Aiken et al. 2003; Foken 2017). Here we use the power law wind profile approximation or the Monin–Obukhov similarity theory with quasi-logarithmic wind profile approximation (Arya 2001; Foken 2017).

### Average prevailing wind direction

In a given geographical location at latitude LA (with 0.125° resolution) and longitude LO (with 0.125° resolution), we used the monthly average values *u*_month_ and *v*_month_ of the *u* and *v* components of the wind speed *w* (Fig. 2) originating from the ERA5 MONTHLY database (Hersbach and Dee 2016; Hersbach et al. 2020). If the hourly measured values of *u* and *v* are *u*_i_ and *v*_i_ (i = 1, 2, …, 23, 24), then the monthly averages of *u*_i_ and *v*_i_ are:

$$u_{{{\text{month}}}} = \frac{{\sum\limits_{{{\text{i}} = 1}}^{N} {u_{{\text{i}}} } }}{N}$$

$$v_{{{\text{month}}}} = \frac{{\sum\limits_{{{\text{i}} = 1}}^{N} {v_{{\text{i}}} } }}{N}$$

1 $$N = { 24}m, m = { 28},{ 3}0,{ 31},$$

where *N* = 24* m* depending on the number *m* = 28, 30 or 31 of days in a given month. We calculated *u*_breed_ and *v*_breed_ being the averages of *u*_month_ and *v*_month_ for May, June, July and August, that is for the breeding-season of sunflowers. Finally, the wind speed components *U* and *V* were calculated, which are the averages of *u*_breed_ and *v*_breed_ for the following four periods: (i) 2014–2023 = 1 decade, (ii) 2004–2023 = 2 decades, (iii) 1974–2023 = 5 decades, (iv) 1940–2023 = 8.3 decades. In a given geographical location, the angle α of the prevailing wind direction measured counter-clockwise from the geographical east averaged for these four periods is (Fig. 2):

2 $$\alpha \, = {\text{ arc tan}}\left( {V/U} \right).$$

### Regional distribution of the average prevailing wind direction

**Fig. 4:**
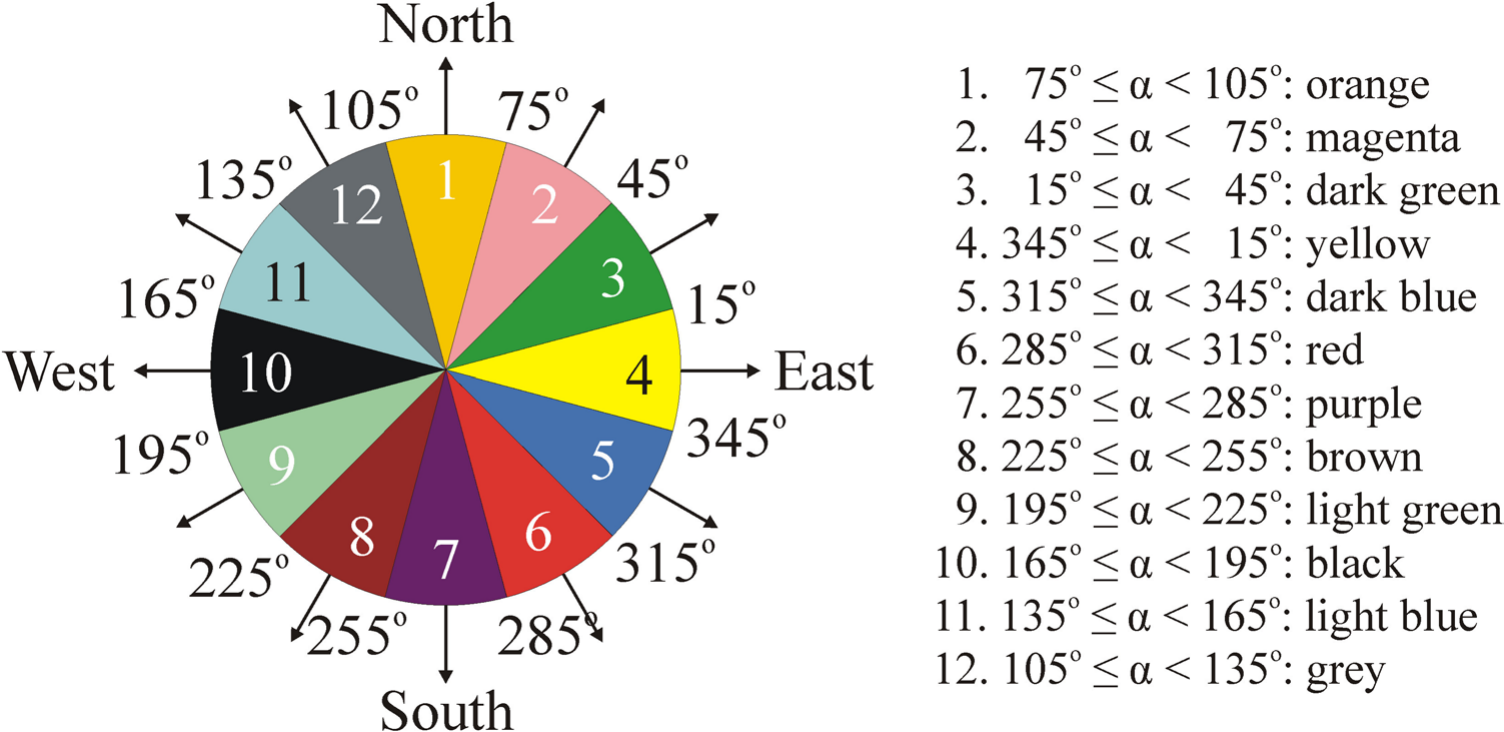
The 360° colour dial defining 12 differently coloured 30°-wide sectors of the wind direction α (marked by arrows) used in the maps of prevailing wind direction in Hungary, Europe and the USA. The eastward pointing yellow sector 4 is the most important for the biomechanical hypothesis tested in this work

The maps of Hungary, Europe and the USA are divided to cells with latitudinal (LA) and longitudinal (LO) angle width LA = 0.125° and LO = 0.125°. For all cells we determined the prevailing wind direction α measured counter-clockwise from geographical east and averaged for the periods 2014–2023, 2004–2023, 1974–2023 and 1940–2023. To define the cell’s colour, the 360° angular interval is divided to 12 differently coloured sectors with an angular width of 30° as shown in Fig. 4.

Thus, we obtained the coloured maps of Hungary, Europe and the USA displaying the regional distribution of the average prevailing wind direction α for the mentioned four averaging periods. Finally, in each coloured map we calculated the area proportion *f* = *N*_colour_/*N*_cells_ of the 12 colours, that is of the 12 different α-intervals, where *N*_colour_ is the number of a given colour (α-interval) and *N*_cells_ is the number of cells in the map. The geographical eastern angular interval −15° ≤ α ≤  + 15°, for example, is yellow (Fig. 4). This eastward pointing 4th sector is the most important for the biomechanical hypothesis tested in this work.

**Fig. 5:**
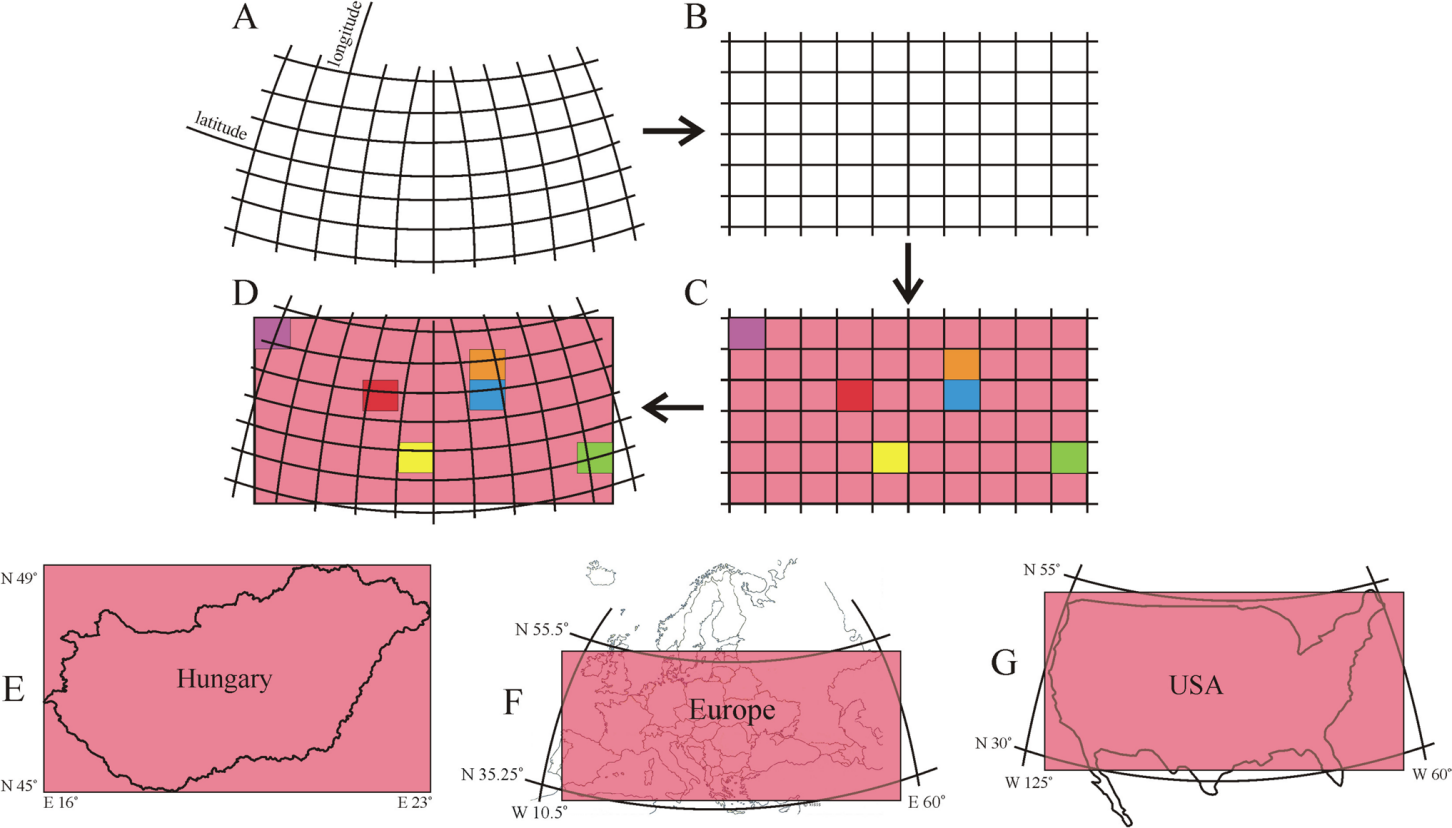
**A** The lines of longitude and latitude in geographical maps are depicted by circular curves. **B** These circles become horizontal and vertical straight lines in the Cartesian coordinate system used by us. **C** The two-dimensional, colour-coded, rectangular distributions of the local prevailing wind directions are displayed in this Cartesian system. **D** Finally, the colour-coded rectangular wind patterns are superposed onto the geographical maps of Hungary, Europe and the USA. **E**, **F**, **G** Maps of the investigated geographical regions of Hungary, Europe and the USA with the extrema of the east(E)-west(W) longitudes and north(N)-south(S) latitudes

In a geographical map, the lines of latitude and longitude are usually circular curves (Fig. 5A). These curves become horizontal and vertical straights in the Cartesian coordinate system (Fig. 5B) used to depict the two-dimensional regional distribution of the colour-coded average prevailing wind direction α (Fig. 5C). Finally, this colour map of α is superposed onto the geographical map (Fig. 5D). Due to this mismatch between the Cartesian (i.e. rectangular) wind pattern and the underlying non-Cartesian (i.e. curved) map, the map informs only approximately about the locality of a given prevailing wind direction. This mismatch is negligible in the case of Hungary because of its small area, the consequence of which is that the longitude and latitude lines are practically straights. Figures 5E–G show the maps of Hungary, Europe and the USA with the extrema of the east (E)–west (W) longitudes and north (N)–south (S) latitudes.

The unfortunate fact is that detailed global or regional databases of sunflower crop lands do not exist. The obvious reason of this is that sunflower plants extremely exhaust the nutrient supply of ploughlands, thus they are not sown every year in the same area. Therefore, sunflowers are grown in crop rotation and their actual crop lands change considerably from year to year. Thus, the exact yearly areas occupied by sunflower crops are not clearly registered, and thus are not available, practically in all countries. Consequently, the most we can do is to estimate the maximal possible area where sunflowers can grow in a given geographical map in such a way that we disregard the areas of larger water surfaces and mountains, where sunflowers cannot be cultivated. In a given geographical map, these disregarded areas are depicted by white colour. In the three studied regions these white areas are the following:

- **Hungary**: lakes Balaton and Fertő; region of capital Budapest; Transdanubium mountains, Northern middle mountains, and Mecsek highland.
- **Europe**: Atlantic ocean; Northern, Baltic, Mediterranean, Black and Caspian seas; lake Aral.
- **USA**: Rocky mountains; Pacific and Atlantic oceans; Gulf of Mexico; Great Lakes and lake Utah.

### Estimation of wind-induced torque on young sunflower heads

**Fig. 6:**
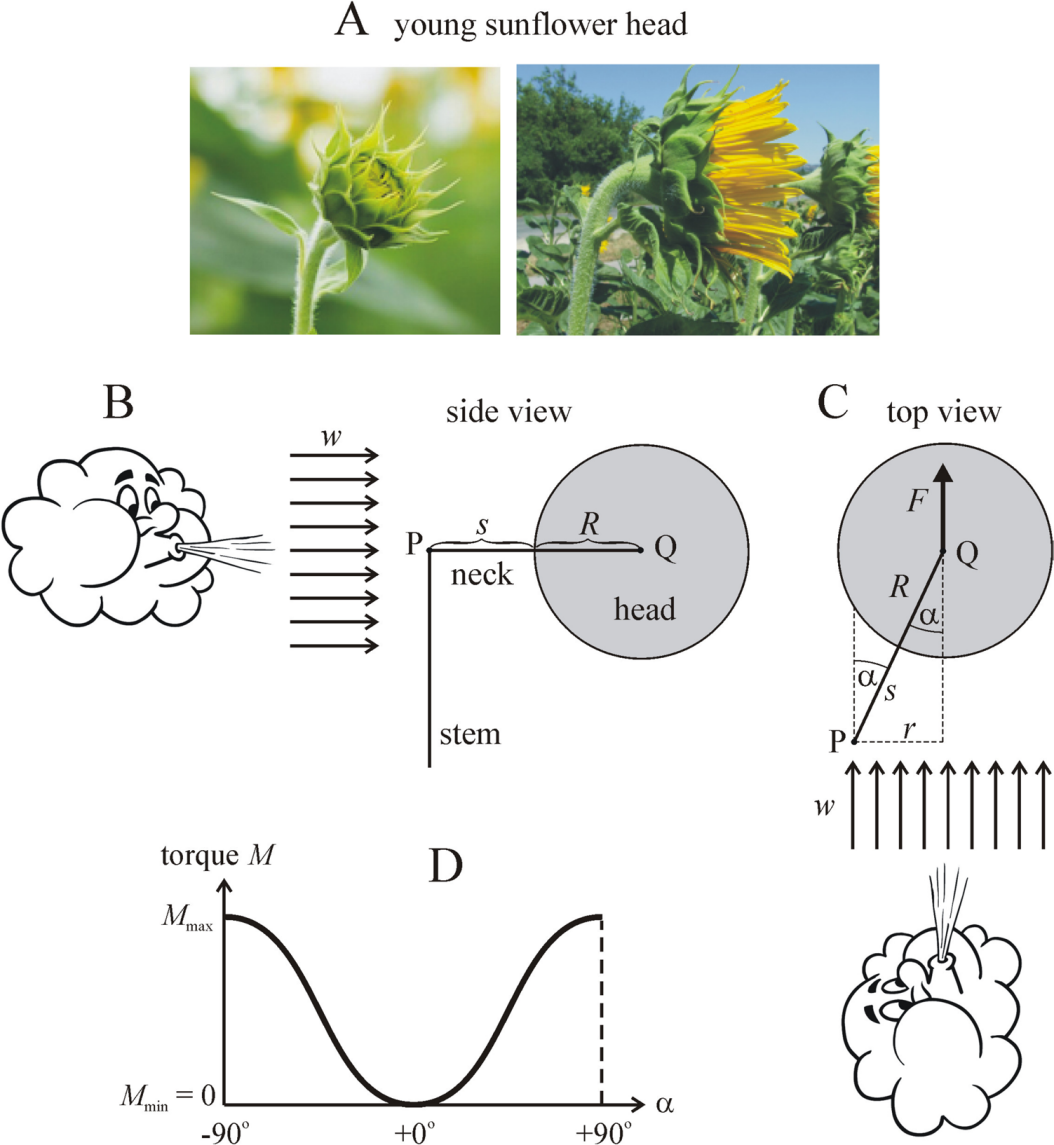
For estimation of wind-induced torque *M* on young sunflower heads. **A** Young sunflower head prior to flowering (photographed by Gábor Horváth). **B** Side view geometry of the model of a wind-blown young sunflower head. The head is modelled by a sphere with radius *R* and center Q. *s*: length of the neck holding the head. P: upper end of the vertical stem. *w*: wind speed. **C** Top view geometry of the model. *F*: air drag force. α: angle of the neck from the wind direction. *r* = (*s* + *R*)·|sinα|. **D** Qualitative change of the wind-induced torque *M* versus angle α

Since young sunflower heads are approximately spherical (Fig. 6A), let us model the young sunflower head by a sphere with radius *R*, which is held by a horizontal neck of length *s*. The neck and the head can turn around the vertical stem due to the wind-induced torque (i.e., turning-moment) *M*. When the head is blown by a wind with speed *w* (Fig. 6B) blowing at angle α relative to the neck (Fig. 6C), then the wind-induced air drag force *F* on the sphere is:

3 $$F = \frac{{kR^{2} \pi \rho_{{{\text{air}}}} w^{2} }}{2},$$

where *k* = 0.2 is the drag form coefficient of a sphere for turbulent flow with Reynold’s number *Re* ≥ 2·10^6^ (Clancy 1975), *R*^2^π is the frontal area of the sphere, ρ_air_ = 1.23 kg/m^3^ is the air density at 15 °C air temperature and sea-level at 1013.25 hPa air pressure, *w* is the wind speed. The drag force is independent of the wind direction α due to rotation symmetry of the sphere. According to Fig. 6C, *r* = (*s* + *R*)·|sinα|, thus the wind-induced torque *M* on the sphere is:

4 $$M = F \cdot r = \frac{{k{\uppi }R^{2} (s + R){\uprho }_{{{\text{air}}}} w^{2} }}{2}\left| {\sin {\upalpha }} \right|$$

## Results

### Wind-induced torque

The torque *M* induced by the prevailing wind could force only young sunflower heads to turn and later keep a constant (e.g. eastward) orientation as assumed by hypothesis (8) mentioned in the Introduction. This torque is estimated by (Eq. 4) with the following numerical values: *k* = 0.2, ρ_air_ = 1.23 kg/m^3^, *R* ≈ 0.05 m, *s* ≈ 0.05 m, where the latter two values are typical averages. Using these values, we obtain from (Eq. 4):

$$M = 9.66 \cdot 10^{ - 5} \cdot w^{2} \cdot \left| {\sin {\upalpha }} \right|$$

5 $$M_{\max } ({\upalpha } = \pm 90^{{\text{o}}} {)} = 9.66 \cdot 10^{ - 5} \cdot w^{2}$$

where the unit of the wind speed is m/s. For a typical average maximum wind speed *w* = 3 m/s (≈ 11 km/h), from (Eq. 5) we obtain the torque’s maximum *M*_max_ = 8.7·10^–4^ Nm. Figure 6D shows qualitatively the change of *M* versus angle α. The torque *M* is maximal at α =  ± 90° and minimal at α = 0° when the neck is perpendicular and parallel to the wind direction, respectively.

During the growing of a sunflower, the torque *M* excerted by the drag force of the prevailing wind on its head tries to turn the neck and head toward the wind direction. This continuous environmental mechanical constraint can be decreased if the stem with the neck and head turns gradually toward the prevailing wind direction α*. When the neck becomes parallel to direction α*, this constraint is eliminated, because then *M* = 0 as seen in Fig. 6D. Although our calculation is only an estimate, expression (Eq. 4) of torque *M* describes qualitatively well the mechanical constraint of the young sunflower heads under windy conditions. We admit however, that the concrete estimated numerical value of the maximal torque *M*_max_ = 8.7·10^–4^ Nm calculated from (Eq. 5) does not help to deepen further the quantification of biomechanical plausibility of hypothesis (8).

### Hungary

**Fig. 7:**
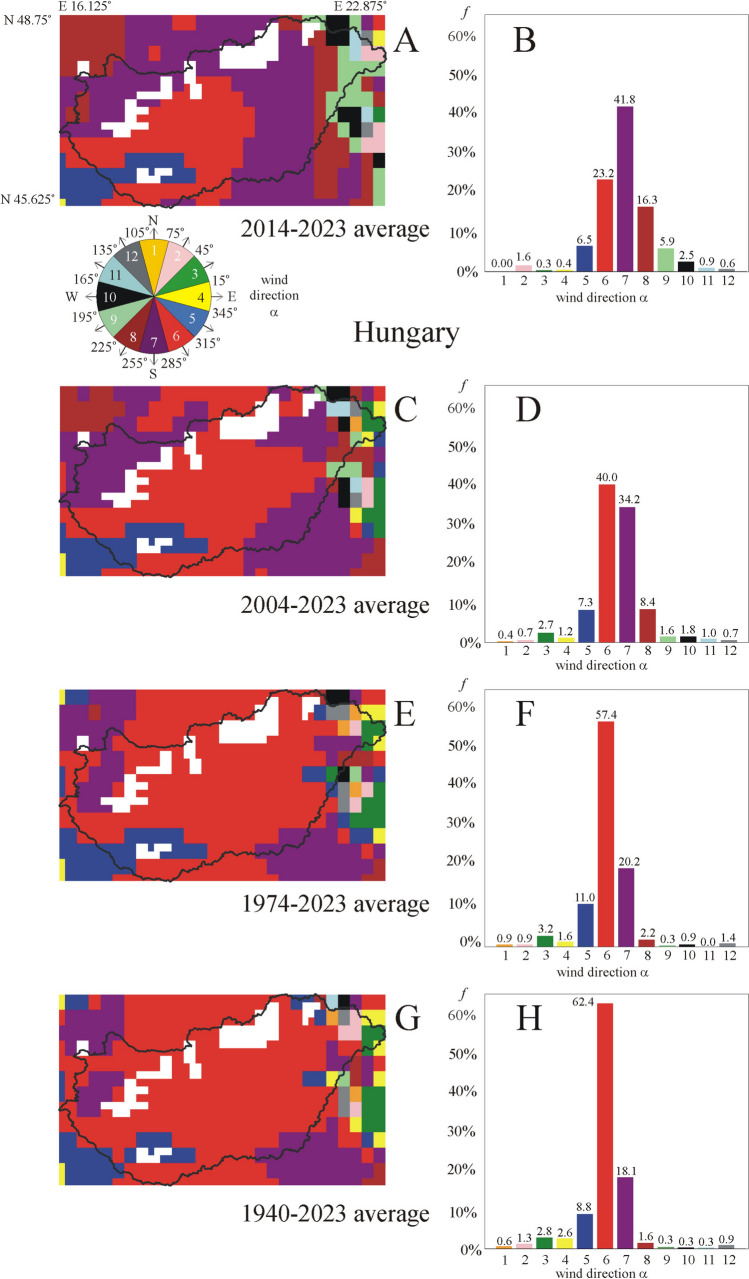
**A**, **C**, **E**, **G** Two-dimensional distributions (maps) of the average prevailing wind direction α in Hungary, where the 12 different colours code the 12 different 30°-wide wind direction intervals shown by the colour dial inset below **A** (see also Fig. 4). The 30°-wide sector pointing to the geographical east α = 0° ± 15°, for example, is yellow and codes winds blowing from nearly west to east. White colour codes areas of larger water surfaces and mountains being inappropriate for sunflower cultivation. **B**, **D**, **F**, **H** Diagrams of the area proportion *f* = *N*_colour_/*N*_cells_, where *N*_colour_ is the number of a given colour (α-interval) and *N*_cells_ is the number of cells in the map. *f* gives the percentage of areas with a given average prevailing wind direction interval relative to the whole area of the rectangular colour pattern with the map of Hungary. The wind data are averaged in the May–August breeding-season of sunflowers for the following periods: 2014–2023 (**A, B**), 2004–2023 (**C**, **D**), 1974–2023 (**E**, **F**), and 1940–2023 (**G**, **H**)

According to Figs. 7A, B, in the sunflower’s May–August breeding-season of the averaging period 2014–2023 the prevailing nearly west-to-east (α = 0° ± 15°, 4. wind colour = yellow) wind direction is characteristic only for *f* = 0.4% of the area of Hungary without larger water surfaces and mountains. The most frequent southern (7. purple) wind direction rules *f* = 41.8% of Hungary, furthermore, the area proportions *f* of south-western (*f* = 16.3%, 8. brown) and south-eastern (*f* = 23.2%, 6. red) wind directions are also large (Fig. 7B).

In case of the averaging period 2004–2023 (Figs. 7C, D), the prevailing eastern (α = 0° ± 15°, 4. yellow) wind direction characterizes only *f* = 1.2% of Hungary, while the southern (*f* = 34.2%, 7. purple) and south-eastern (*f* = 40%, 6. red) wind directions have the largest area proportion *f* (Fig. 7D).

Averaging for the period 1974–2023 (Figs. 7E, F), the prevailing eastern (α = 0° ± 15°, 4. yellow) wind direction dominates only *f* = 1.6% of Hungary. The most frequent wind directions are south-eastern (*f* = 57.4%, 6. red; *f* = 11%, 5. dark blue) and southern (*f* = 20.2%, 7. purple) (Fig. 7F).

Considering the averaging period 1940–2023 (Figs. 7G, H), the prevailing eastern (α = 0° ± 15°, 4. yellow) wind direction is typical only for *f* = 2.6 ± 5.8% of Hungary. The most frequent south-eastern (6. red) and southern (7. purple) wind directions rule *f* = 62.4% and *f* = 18.1% of Hungary, respectively (Fig. 7H).

In sum, the southern and south-eastern wind directions dominate Hungary in the May–August breeding-season of sunflowers, while the eastern wind direction is characteristic only for smaller than *f* = 2.6 ± 5.8% of the area of Hungary without larger water surfaces and mountains.

### Europe

**Fig. 8:**
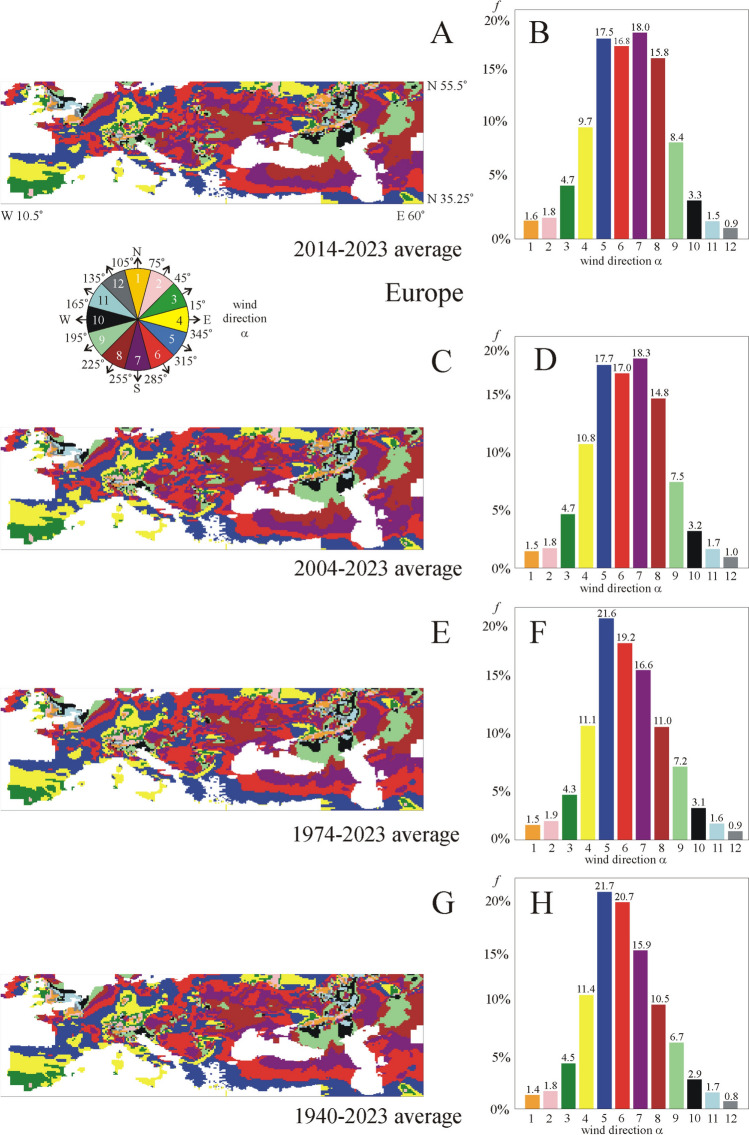
Same as Fig. 7 for Europe

Figures 8A, B show that in the averaging period 2014–2023, eastern (α = 0° ± 15°, 4. yellow) wind direction dominates only *f* = 9.7% of the area of Europe without larger water surfaces and mountains, mainly in the south-west and north regions. South-eastern (5. dark blue, 6. red), southern (7. purple) and south-western (8. brown) wind directions rule *f* = 68% of Europe (Fig. 8B), which means that the average prevailing wind directions differed by −15° to −135° from the eastern (α = 0°) direction.

Averaging for the period 2004–2023 (Figs. 8C, D), the eastern (α = 0° ± 15°, 4. yellow) wind direction characterizes only *f* = 10.8% of Europe. Then, in the majority of Europe, south-eastern and southern wind directions dominate.

Considering the averaging period 1974–2023 (Figs. 8E, F), eastern (α = 0° ± 15°, 4. yellow) wind direction rules only *f* = 11.1% of Europe. South-eastern (5. dark blue, 6. red) and southern (7. purple) wind directions are typical for *f* = 21.6%, 19.2% and 16.6% of the continent, respectively (Fig. 8F).

In case of the averaging period 1940–2023 (Figs. 8G, H), eastern (α = 0° ± 15°, 4. yellow) wind direction dominates only *f* = 11.4 ± 5.0% of Europe, predominantly the south-west, north and middle-west regions. South-eastern (5. dark blue and 6. red) wind directions characterize *f* = 42.4% of the continent (Fig. 8H).

Hence, depending on the averaging period, eastern (α = 0° ± 15°, 4. yellow) wind direction rules only *f* = 9.7–11.4% of the area of Europe between N 35.25° and N 55.5° latitudes and W 10.5° and E 60° longitudes without larger water surfaces and mountains.

### USA

**Fig. 9:**
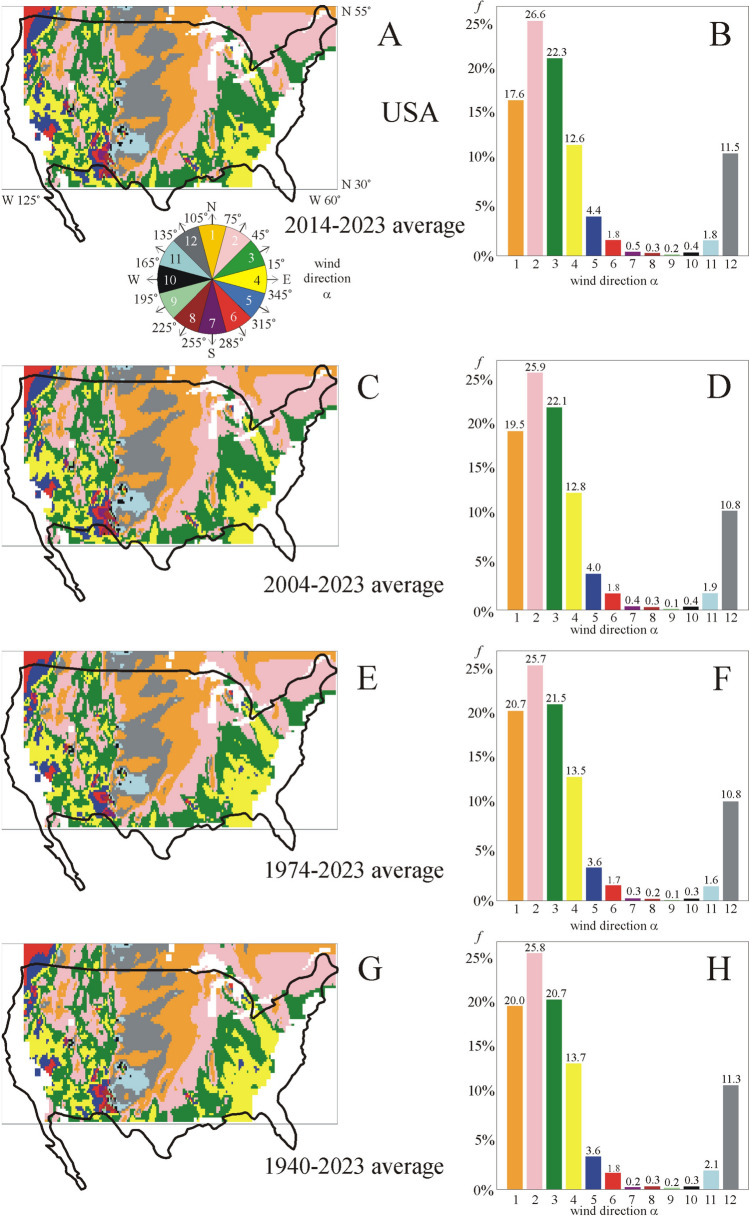
Same as Fig. 7 for the USA

According to Figs. 9A, B, in the averaging period 2014–2023, the eastern (α = 0° ± 15°, 4. yellow) wind direction rules only 12.6% of the area of USA without larger water surfaces and mountains, mainly in the east, south and west regions, forming an U-shape. In this U-shaped region the most frequent prevailing winds blow toward north-east with *f* = 26.6% (2. magenta) and *f* = 22.3% (3. dark green) area proportion. In the middle north–south zone of USA, between the two branches of the U-shape, northern (1. orange, *f* = 17.6%) and north-western (12. grey, *f* = 11.6%) prevailing winds dominate (Figs. 9A, B).

In case of the averaging periods 2004–2023 (Figs. 8C, D), 1974–2023 (Figs. 9E, F) and 1940–2023 (Figs. 9G, H), the eastern (α = 0° ± 15°, 4. yellow) wind direction is typical only for *f* = 12.8%, 13.5% and 13.7 ± 2.8% of the USA, respectively, and all features of the wind pattern remain qualitatively and practically the same as for the period 2014–2023.

In sum, practically independently of the averaging period, in the area of the USA without larger water surfaces and mountains the followings are true for the prevailing wind directions: (i) The middle north–south zone of the country is ruled by northern and north-western winds; (ii) in the U-shaped west, south and east regions of the country, eastern and north-eastern wind directions are typical; (iii) eastern (α = 0° ± 15°) wind direction dominates only *f* = 12.6–13.7% of the country.

## Discussion and conclusions

The many physiological details of sunflower heliotropism/phototropism, nocturnal reorientation and circadian regulation revealed in the last decades (Shell et al. 1974; Shell and Lang 1976; Blackman et al. 2011; Sabetta et al. 2011; Kane et al. 2013; Mandel et al. 2013; Vandenbrink et al. 2014; Atamian et al. 2016; Kutschera and Briggs 2016) highlight the complexity of the orientation behaviour of *Helianthus annuus*. In the Introduction we listed the eight existing hypotheses which try to explain the possible benefits of the east facing of mature sunflower inflorescences. Let us first briefly summarize the status of these hypotheses.

According to **hypothesis (1)**, non-skyward orientation may be advantageous, because the zone on tilted sunflower heads is narrower where granivorous birds can cling (Seiler 1997). Since this explanation is directionally non-selective and holds true for any azimuth direction of the head, it does not explain at all the east facing of sunflower inflorescences.

According to **hypothesis (2)**, the eastward orientation of sunflower inflorescences may have the advantage that the heat stress of the head is reduced near noon (Leshem 1977; Lang and Begg 1979). However, a fixed westerly orientation would have the same advantage.

According to **hypothesis (3)**, the eastward orientation of sunflower inflorescences may ensure that the heads absorb much amount of light in the early morning, which may speed up drying of morning dew and thus could reduce the risk of fungal attack (Lang and Begg 1979). Although this idea seems to be sound, it has not been tested experimentally yet.

According to **hypothesis (4)**, the east facing of sunflowers could reduce heat load, especially in the afternoon with high irradiance (Seiler 1997). Lower inflorescence temperatures may increase the crop yield by improving the viability and fertility of pollens, or improving grain filling during seed development (Ploschuk and Hall 1995; Seiler 1997). However, in afternoons the air temperature, rather than the solar irradiance, is usually higher than in mornings (Noel et al. 2018).

According to **hypothesis (5)**, higher head temperatures result in more rapid seed maturation and reduced grain filling (Ploschuk and Hall 1995). Since near noon the temperature of east-facing sunflower inflorescences is smaller by 3–8 °C than that of heads turned artificially toward the zenit (Lang and Begg 1979; Lamprecht et al. 2007), eastward orientation could increase the seed weight. However, due to temporal symmetry, the same is also true for westward oriented sunflower heads.

As mentioned in the Introduction, **hypothesis (6)** was refuted by the findings of field experiments (Horváth et al. 2024), **hypothesis (7)** was corroborated by computational results based on astronomical, meteorological and plant physiological data (Horváth et al. 2020). Finally, **hypothesis (8)** is refuted by the results of the present paper.

In this work, we tested the most recent biomechanical hypothesis (8) that the constant eastward orientation of mature sunflower inflorescences could be caused by the torque of the local prevailing wind blowing from west to east. In this case the air drag would turn the sunflower head’s normal vector always eastward, like the weathervane/cock is turned parallel to the wind direction (Fig. 1). Since mature sunflower inflorescences face nearly east all over the world, this hypothesis would be validated only, if the local prevailing wind direction were west-to-east all over the cultivation area of sunflowers. Using the ERA5 MONTHLY wind dataset, we determined the area proportion *f* of the local prevailing eastward α = 0° ± 15° blowing wind in Hungary, Europe and the USA averaged for the May–August breeding-season of sunflowers and for four different periods of 1 (2014–2023), 2 (2004–2023), 5 (1974–2023) and 8.3 (1940–2023) decades. Our studies were restricted to those northern latitudes (whole Hungary: 45.625°–48.75°; Europe: 35.25°–55.5°; USA: 30°–55°) where the climate is warm enough for sunflower growing. From our investigation we omitted the areas of larger water surfaces and mountains where sunflowers cannot be grown.

Although by this we overestimated the actual cultivation regions of sunflowers, this is practically the maximum what we can do, because there are not exact up-to-date maps about the real areas of sunflower cultivation. The main reason of this is that the areas planted by sunflower change yearly because of crop rotation. The most detailed data about sunflower cultivations are available from FAOSTAT: Food and Agriculture Organization Corporate Statistical Database (https://www.destatis.de/EN/Themes/Economic-Sectors-Enterprises/Agriculture-Forestry-Fisheries/Field-Crops-Grassland/Tables/arable-land-after-the-main-groups-and-crops.html) and USDA: United State Department of Agriculture (https://www.ers.usda.gov/data-products/charts-of-note/chart-detail?chartId=110335). However, from these databases only the yearly acreages under sunflower cultivation can be gained. For example, in the last decade in Europe the minimum (in 2016) and maximum (in 2022) sunflower areas per 1000 hectare were 16.7 and 85.6 acres, respectively. On the other hand, in the USA, the minimum (2025) and maximum (2000) planted and harvested sunflower acres are 750 000 and 2 800 000 acres, respectively.

These and similar acreage data do not help to reveal the precise regional distribution (maps) of sunflower cultivations. However, this is not a serious problem considering our main conclusion that mature sunflower inflorescences face east practically independently of the prevailing wind direction. If the frequency *f* of the 12 different categories of the prevailing wind direction α in Hungary, Europe and the USA were uniform, then every α would have the same frequency *f* = 100/12 = 8.3%. From Figs. 7, 8 and 9 it is clear that this is not true. There are large differences in *f* between the different α-values. The percentages 2.6 ± 5.8%, 11.4 ± 5.0% and 13.7 ± 2.8% of regions with average eastern prevailing wind direction in Hungary, Europe and the USA are so small that alone these tiny percentages make obvious that the wind direction (being not everywhere eastern) cannot explain the fact that all mature sunflower inflorescences face east all over the world.

Beyond the area proportion *f* of the local prevailing eastward (α = 0° ± 15°) wind direction, we also determined *f* of 11 other prevailing direction intervals (α = x^o^ ± 15°, x = 30°, 60°, 90°, 120°, 150°, 180°, 210°, 240°, 270°, 300°, 330°) of wind blowing in all directions of the wind rose (Fig. 4). We found the following:

o In Hungary—between northern latitudes 45.625° and 48.75°, and eastern longitudes 16.125° and 22.875° (Fig. 7)—depending on the averaging period, the prevailing eastern (α = 0° ± 15°) wind direction characterizes only *f* = 0.4–2.6% of the country’s area without larger water surfaces and mountains, mainly in the eastern and western regions near the national border. For the averaging period 2014–2023, the most dominating wind direction is south ± 15°. Increasing the averaging period up to 1940–2023, the prevailing wind direction turns toward south-east ± 15°, and the area proportion of the eastern (α = 0° ± 15°) wind direction increases from *f* = 0.4% to *f* = 2.6%.
o In Europe—between northern latitudes 35.25° and 55.5°, and longitudes E 60° and W 10.5° (Fig. 8)—depending on the averaging period, the prevailing eastern (α = 0° ± 15°) wind direction rules only *f* = 9.7–11.4% of the continent without larger water surfaces and mountains. This is true predominantly for the south-west and sporadically the north regions.
o In the USA—between northern latitudes 30° and 55°, and western longitudes 60° and 125° (Figs. 9)—depending only slightly on the averaging period, the prevailing eastern (α = 0° ± 15°) wind direction dominates only *f* = 12.6–13.7% of the country without larger waters and mountains. In the middle of the country, northern and north-western prevailing wind directions are typical, while the west, south and east regions are characterized by eastern and north-eastern wind directions.

On the basis of the wind data averaged for the longest available period 1940–2023 and the sunflower’s May–August breeding-season, we conclude that the local prevailing eastern (α = 0° ± 15°) wind direction rules only *f* = 2.6 ± 5.8%, 11.4 ± 5.0% and 13.7 ± 2.8% of Hungary, Europe and the USA, respectively, without larger water surfaces and mountains. Hence, the prevailing local eastward ± 15° wind could explain/cause the east facing of mature sunflower inflorescence only in these small area proportions in Hungary, Europe and the USA. Since mature sunflower inflorescences orient constantly nearly to the geographical east all over the world (Vandenbrink et al. 2014; Atamian et al. 2016; Takács et al. 2022a), our results do not support hypothesis (8) mentioned in the Introduction, because its meteorological prerequisite is not fulfilled. On the other hand, where mature sunflower inflorescences do not face east, there this rare effect is caused by the special illumination conditions. For example, when the direct sunlight is restricted by shadings to a given time of day between sunrise and sunset (Horváth et al. 2020; Takács et al. 2022a).

In principle, the east facing of mature sunflower inflorescences could be more directly tested by experimental treatments with controls to dissect the contributions of wind and light directions. However, our finding makes unnecessary such an experimental fieldwork. On the one hand, the torque exerted by wind on the sunflower head could be studied in a wind-tunnel. In this case, the physically trivially expected effect is that this torque turns the normal vector of the head toward the wind direction, as visualized in Fig. 1. Since in a wind-tunnel the natural light conditions (temporally changing direct sunlight and diffuse scattered skylight) cannot be ensured, this approach would be inappropriate to reveal the relative contributions of wind and light directions to the sunflower’s east facing. On the other hand, individual sunflowers growing in the open air could be continuously blown by artificial winds (produced by fans) with different directions during the breeding-period. Only such a time-consuming and logistically difficult field experiment could guarantee the simultaneous influence of wind and natural illumination on growing sunflowers. However, such an investigation would be completely unnecessary, because the wind results presented in this work evidently show that the effect of natural light conditions on sunflowers overwhelms the effect of the wind direction. We showed that sunflower inflorescences orient to east not only in regions with eastward blowing prevailing wind, but also in regions where the prevailing wind direction considerably differs from the east direction. Hence, our finding that all sunflower inflorescences face east independently of the prevailing wind direction during the breeding-season proves that the effect of wind direction is an unimportant environmental factor and the light conditions govern predominantly the final orientation of mature sunflower inflorescences.

## Data Availability

All data underlying the results presented are available in this paper.

## References

[CR1] Aiken RM, Nielsen DC, Ahuja LR (2003) Scaling effects of standing crop residues on the wind profile. Agron J 95:1041–1046. 10.2134/agronj2003.1041

[CR2] Arya SP (2001) Introduction to micrometeorology, 2nd edn. Elsevier, Academic Press, Cambridge

[CR3] Atamian HS, Creux NM, Brown EA, Garner AG, Blackman BK, Harmer SL (2016) Circadian regulation of sunflower heliotropism, floral orientation, and pollinator visits. Science 353:587–590. 10.1126/science.aaf9793 27493185 10.1126/science.aaf9793

[CR4] Blackman BK, Scascitelli M, Kane NC, Luton HH, Rasmussen DA, Bye RA, Lentz DL, Rieseberg LH (2011) Sunflower domestication alleles support single domestication center in eastern North America. Proc Natl Acad Sci U S A 108:14360–14365. 10.1073/pnas.1104853108 21844335 10.1073/pnas.1104853108 PMC3161615

[CR5] Clancy LJ (1975) Aerodynamics. Pitman Publishing Limited, London

[CR6] Darwin C, Darwin F (1897) The power of movement in plants. D. Appleton & Co, New York

[CR7] FAOSTAT https://www.destatis.de/EN/Themes/Economic-Sectors-Enterprises/Agriculture-Forestry-Fisheries/Field-Crops-Grassland/Tables/arable-land-after-the-main-groups-and-crops.html

[CR8] Foken T (2017) Micrometeorology. Springer, Heidelberg, Berlin, New York

[CR9] Hersbach H, Dee D (2016) ERA5 reanalysis is in production. ECMWF Newslett 147:7

[CR10] Hersbach H, Bell B, Berrisford P, Hirahara S, Horanyi A et al (2020) The ERA5 global reanalysis. Q J R Meteorol Soc 146:1999–2049. 10.1002/qj.3803

[CR11] Horváth G, Slíz-Balogh J, Horváth Á, Egri Á, Virágh B, Horváth D, Jánosi IM (2020) Sunflower inflorescences absorb maximum light energy if they face east and afternoons are cloudier than mornings. Sci Rep 10:21597. 10.1038/s41598-020-78243-z 33299003 10.1038/s41598-020-78243-z PMC7725789

[CR12] Horváth G, Dárdai B, Bíró M, Slíz-Balogh J, Száz D, Barta A, Egri Á (2024) The all-day pollinator visits of sunflower inflorescences in *Helianthus annuus* plantations are independent of head orientation: testing a wide-spread hypothesis. Plant J 120:1563–1576. 10.1111/tpj.17070 39395022 10.1111/tpj.17070

[CR13] Horváth G (2023) Photobiophysics of *Helianthus annuus*: why do mature sunflower inflorescences face geographical east? (Lecture) In: Satellite Section: 25. jubilee meeting of the Department of Biological Physics of the Eötvös Loránd University on the 29. Congress of the Hungarian Biophysical Society, Budapest, 31 August 2023, 14:00–14:20. https://mbft.hu/kongresszus2023/, https://mbft.hu/kongresszus2023/program.php.

[CR14] Kane NC, Burke JM, Marek L, Seiler G, Vear F, Baute G, Knapp SJ, Vincourt P, Rieseberg LH (2013) Sunflower genetic, genomic and ecological resources. Mol Ecol Resour 13:10–20. 10.1111/1755-0998.12023 23039950 10.1111/1755-0998.12023

[CR15] Kutschera U, Briggs WR (2016) Phototropic solar tracking in sunflower plants: an integrative perspective. Ann Bot 117:1–8. 10.1093/aob/mcv141 26420201 10.1093/aob/mcv141 PMC4701145

[CR16] Lamprecht I, Maierhofer C, Röllig M (2007) Infrared thermography and thermometry of phototropic plants. J Therm Anal Calorim 87:49–54. 10.1007/s10973-006-7806-9

[CR17] Lang ARG, Begg JE (1979) Movements of *Helianthus annuus* leaves and heads. J Appl Ecol 16:299–305. 10.2307/2402749

[CR18] Leshem YY (1977) Sunflower: a misnomer? Nature 269:102. 10.1038/269102d0

[CR19] Mandel JR, Nambeesan S, Bowers JE, Marek LF, Ebert D, Rieseberg LH, Knapp SJ, Burke JM (2013) Association mapping and the genomic consequences of selection in sunflower. PLoS Genet 9:e1003378. 10.1371/journal.pgen.1003378 23555290 10.1371/journal.pgen.1003378 PMC3605098

[CR20] Noel V, Chepfer H, Chiriaco M, Yorks J (2018) The diurnal cycle of cloud profiles over land and ocean between 51° S and 51° N, seen by the CATS spaceborne lidar from the International Space Station. Atmos Chem Phys 18:9457–9473. 10.5194/acp-18-9457-2018

[CR21] Ploschuk EL, Hall AJ (1995) Capitulum position in sunflower affects grain temperature and duration of grain filling. Field Crops Res 44:111–117. 10.1016/0378-4290(95)00079-8

[CR22] Rajna E (2024) Where could the local prevailing wind direction cause the geographical eastward orientation of mature sunflower inflorescences? Testing a biomechanical hypothesis using Hungarian, European and North-American wind data. B.Sc. diploma work, Department of Biological Physics, Eötvös University, Budapest, Hungary, p. 47 (supervisor: Horváth G.)

[CR23] Sabetta W, Alba V, Blanco A, Montemurro C (2011) Suntill: a tilling resource for gene function analysis in sunflower. Plant Methods 7:20. 10.1186/1746-4811-7-20 21718494 10.1186/1746-4811-7-20 PMC3169506

[CR24] Seiler GJ (1997) Anatomy and morphology of sunflower. In: Schneiter AA (ed) Sunflower technology and production. American Society of Agronomy, Madison, pp 67–111

[CR25] Shell GSG, Lang ARG (1976) Movements of sunflower leaves over a 24-h period. Agric Meteorol 16:161–170

[CR26] Shell GSG, Lang ARG, Sale PJM (1974) Quantitative measures of leaf orientation and heliotropic response in sunflower, bean, pepper and cucumber. Agric Meteorol 13:25–37

[CR27] Stull RB (1988) An introduction to boundary layer meteorology. Springer, Heidelberg, Berlin, New York

[CR28] Takács P, Kovács Z, Száz D, Egri Á, Bernáth B, Slíz-Balogh J, Nagy-Czirok M, Lengyel Z, Horváth G (2022a) Mature sunflower inflorescences face geographical east to maximize absorbed light energy: orientation of *Helianthus annuus* heads studied by drone photography. Front Plant Sci 13:842560. 10.3389/fpls.2022.842560 35371122 10.3389/fpls.2022.842560 PMC8969559

[CR29] Takács P, Slíz-Balogh J, Száz D, Horváth G (2022b) East-facing *Helianthus annuus* has maximal number and mass of kernel-filled seeds: seed traits versus head orientation. Plant-Environ Inter 3:130–139. 10.1002/pei3.10083 10.1002/pei3.10083 PMC10168033 37284427

[CR30] USDA https://www.ers.usda.gov/data-products/charts-of-note/chart-detail?chartId=110335

[CR31] Vandenbrink JP, Brown EA, Harmer SL, Blackman BK (2014) Turning heads: the biology of solar tracking in sunflower. Plant Sci 224:20–26. 10.1016/j.plantsci.2014.04.006 24908502 10.1016/j.plantsci.2014.04.006

